# Griffithsin, a Highly Potent Broad-Spectrum Antiviral Lectin from Red Algae: From Discovery to Clinical Application

**DOI:** 10.3390/md17100567

**Published:** 2019-10-06

**Authors:** Choongho Lee

**Affiliations:** College of Pharmacy, Dongguk University, Goyang 10326, Korea; lkj640@gmail.com; Tel.: +82-31-961-5223

**Keywords:** griffithsin (GRFT), lectin, carbohydrate-binding, human immunodeficiency virus (HIV), microbicide, virus entry inhibitor

## Abstract

Virus entry into a susceptible host cell is the first step in the formation of all viral diseases. Controlling viral infections by disrupting viral entry is advantageous for antibody-mediated neutralization by the host’s immune system and as a preventive and therapeutic antiviral strategy. Recently, several plant-derived carbohydrate-binding proteins (lectins) have emerged as a new class of antiviral biologics by taking advantage of a unique glycosylation pattern only found on the surface of viruses. In particular, a red algae-derived griffithsin (GRFT) protein has demonstrated superior in vitro and in vivo antiviral activity with minimum host toxicity against a variety of clinically relevant, enveloped viruses. This review examines the structural characteristics of GRFT, focusing on its carbohydrate-binding capability. Its in vitro antiviral profiles against human immunodeficiency virus (HIV) are also discussed followed by a description of the results from a combination study using anti-HIV drugs. The results of several studies regarding its novel antiviral mechanism of action are provided in conjunction with an explanation of viral resistance profiles to GRFT. In addition, its in vitro and in vivo host toxicity profiles are summarized with its pharmacokinetic behavior using in vivo efficacy study results. Also, a large-scale production and formulation strategy, as well as a drug delivery strategy, for GRFT as a new class of broad-spectrum microbicides is discussed. Finally, results from two ongoing clinical studies examining GRFT’s effects on viruses are presented.

## 1. Introduction

Every virus starts its life cycle by entering a susceptible host cell. The host cell-targeting ability of a virus is mainly determined by the presence of appropriate host cell receptors which are engaged by a virus. For successful host cell entry, enveloped viruses have evolved a diverse array of envelope glycoproteins on their membrane with different receptor association capabilities. The best-characterized example of this viral glycoprotein–host cell receptor relationship might be the interaction between the human immunodeficiency virus (HIV) glycoprotein gp120 and the human T lymphocyte CD4 molecule. Due to its essential role in the overall virus life cycle, viral entry has been an attractive target for both vaccine and antiviral drug development with the goal of disrupting the binding of viral glycoproteins to host cell receptors. In addition, heavy and unique glycosylation patterns found only on viral glycoproteins provide another level of virus specificity, which could increase the selectivity of virus-targeting therapeutics and prophylactics. In this regard, the use of carbohydrate-binding proteins, collectively called “lectins”, has been explored as a new therapeutic antiviral strategy. Recently discovered virus-targeting lectins include banana lectin [1], cyanovirin [2], microvirin [3], scytovirin [4], and griffithsin (GRFT) [5]. Among them, GRFT has been studied extensively for the development of either antiviral therapeutics or preventive microbicides due to its extreme potency, low in vitro and in vivo host toxicity, and favorable subclinical outcomes. 

Serious endeavors to develop antiviral therapeutics and vaccines against HIV began during the epidemics of the early 1980s. HIV infection was linked with the development of acquired immune deficiency syndrome (AIDS). With no effective antiviral drugs available during that time, a diagnosis of HIV infection was a death sentence for most patients [6]. However, the introduction of highly active antiretroviral therapy (HAART) improved patient outcomes, rendering AIDS as a clinically manageable disease. However, a lack of preventive measures such as an HIV vaccine has hindered efforts to decrease the number of new HIV cases per year. Particularly, young women who live in resource-poor and HIV-endemic regions such as sub-Saharan Africa [7] have been the most vulnerable population to new HIV infections since they do not have the freedom to control sexual situations or use protective devices. Individuals who practice unprotected receptive anal intercourse (URAI) are also responsible for the ongoing spread of new HIV infections because of a high preponderance of HIV target cells in the traumatized rectal mucosal area [8]. Therefore, these two HIV-vulnerable populations need new types of HIV prevention strategies. To that end, topical anti-HIV microbicides such as creams, gels, or intravaginal rings could be promising, with on-demand pre-exposure prophylactic options not only for vulnerable populations but also for the general public. 

**Figure 1:**
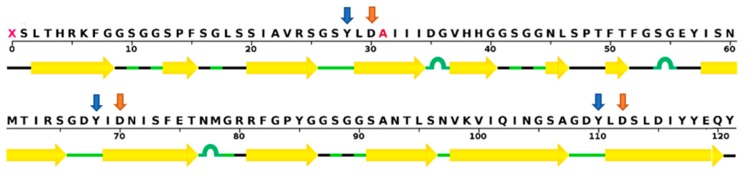
The amino acid sequence of griffithsin (GRFT). Corresponding secondary β-sheet structures are marked with yellow arrows based on the crystal structure of a GRFT dimer with mannose at a resolution of 1.78 Å [19]. Tyrosine and aspartate residues in three carbohydrate-binding domains, which are essential for mannose-binding, are noted with blue and orange arrows, respectively. Wild-type GRFT has a non-standard amino acid at position 31, but it is replaced by alanine (colored in red) in recombinant GRFT. The sequence display was generated by the RCSB protein data bank website (www.rcsb.org).

**Figure 2:**
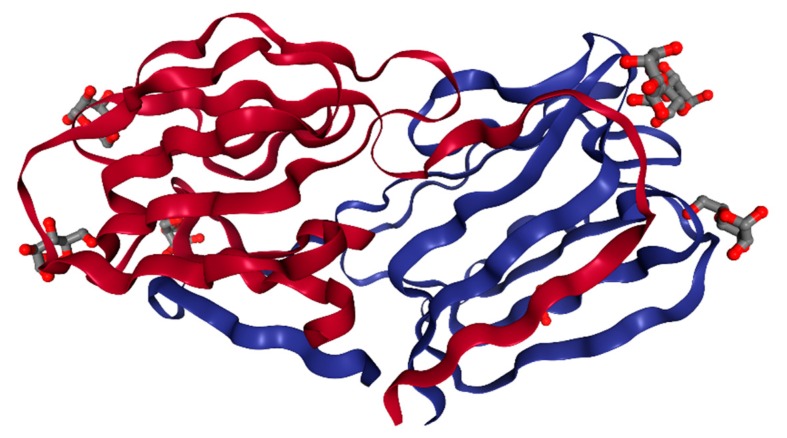
The crystal structure of a GRFT dimer with six mannoses at a resolution of 1.78 Å [19]. Each GRFT monomer is presented in either red or blue. Six bound mannose molecules are shown with ball and stick models. This image was created with PDB ID and associated publication, NGL Viewer (AS Rose et al. (2018) NGL viewer: web-based molecular graphics for large complexes. Bioinformatics doi:10.1093/bioinformatics/bty419), and RCSB PDB.

GRFT is a red algae-derived lectin of 121 amino acids (Figure 1). It exhibits potent (EC_50_ in the picomolar range) and broad-spectrum antiviral activity and negligible in vitro and in vivo host toxicity [9]. Its antiviral activity relates to a unique structural feature that forms a homodimeric complex with three carbohydrate-binding domains on each monomer (Figure 2). These three carbohydrate-binding domains target high-mannose arrays present on many pathogenic enveloped viruses including HIV; severe, acute, or Middle East respiratory syndrome coronaviruses (SARS-CoV or MERS-CoV) [10,11]; hepatitis C virus (HCV) [12,13]; herpes simplex virus 2 (HSV-2) [14,15]; Japanese encephalitis virus (JEV) [16,17]; and porcine epidemic diarrhea virus (PEDV) [18]. As a result of its broad antiviral spectrum, it shows great promise as a general microbicidal agent that can prevent viral transmission and as a therapeutic against enveloped virus-mediated diseases. From discovery to clinical development, much interest has surrounded GRFT because of its potential to be the first clinically proven, preventive measure against various viral diseases. In this review, the structural characteristics of GRFT are examined. Also, its in vitro antiviral activity against HIV, which is the most studied target pathogen for GRFT so far, is discussed in detail using a variety of combination study results involving current antiretroviral drugs. The antiviral mechanism of action for GRFT is explained in conjunction with viral resistance profiles to GRFT. In addition, its in vitro and in vivo toxicity profiles are summarized, and its pharmacokinetic and in vivo efficacy study results are delineated. To provide a practical drug development perspective, a large-scale production and formulation strategy as well as a drug delivery strategy for GRFT as an HIV-preventive biologic is discussed. Finally, ongoing clinical studies of GRFT and its effects on viruses other than HIV are summarized. 

## 2. Structure

GRFT is a carbohydrate-binding protein made of 121 amino acids and is 12.7 kDa in size [9]. It has a unique amino acid residue at position 31 which does not match any of the 20 standard amino acids [5] (Figure 1). It was initially isolated from an aqueous extract of the red algae *Griffithsia* sp. collected from waters off the shores of New Zealand. Researchers at the U.S. National Cancer Institute first reported its potent cytoprotective activity against HIV-1 in T-lymphoblastoid cells [5]. Five research papers reported structural results on GRFT by using either X-ray crystallography or nuclear magnetic resonance (NMR) techniques (Table 1) [19,20,21,22,23]. In terms of structural classification, GRFT is a Jacalin-related lectin harboring three repeats of an antiparallel four-stranded β-sheet with a triangular prism shape (Figure 2) [19]. It is called a domain-swapped dimer because two β-strands from one protein forming the dimer display domain-exchanging properties with the same two β-strands of its counterpart [19]. It has three identical carbohydrate-binding sites (located within residues 20–34, 58–76, and 96–120) on each monomer with three conserved glycine-rich repeats (GGSGG) [19]. It is estimated that as many as 11 high-mannose oligosaccharides are present on one HIV-1 gp120 protein. Therefore, GRFT’s multivalent interactions with gp120 via these three carbohydrate-binding domains seem to be essential for its high-affinity and anti-HIV-1 potency at low concentrations (picomolar range) [22]. In particular, three aspartate residues in these carbohydrate-binding sites (Asp30, Asp70, and Asp112) play a critical role in the interaction of GRFT with high-mannose type oligosaccharides such as Man9GlcNAc2, which is composed of nine mannose molecules and two *N*-acetyl glucosamines [22]. This was supported by a study demonstrating that GRFT point mutations (D30A, D70A, or D112A) partially inhibit its ability to bind to gp120, and a GRFT mutant with all three mutations loses almost all of its binding capacity [21]. In addition, tyrosine residues such as Tyr28, Tyr68, and Tyr110 are necessary for GRFT to bind to carbohydrates [24]. However, the apparent disparity between the gp120-binding ability and HIV-1 inhibitory potency for these GRFT variants indicates the existence of another antiviral mechanism beyond simple gp120-GRFT binding [21]. With regard to carbohydrate-binding specificity, all six GRFT dimer carbohydrate-binding sites can be occupied by mannose, glucose, *N*-acetyl glucosamine, and 1-6 α-mannobiose with similar affinities [23]. To determine whether GRFT must form a dimer to exert its antiviral activity, a monomeric form of GRFT (mGRFT) was created by inserting either two or four amino acids at its dimerization interface. mGRFT possesses greatly reduced antiviral activity against HIV-1 in spite of its comparable association with high-mannose oligosaccharides, since mGRFT possesses all three carbohydrate-binding sites [20]. This suggests that the intact dimeric form of GRFT is required to efficiently disrupt HIV-1 infectivity [20].

## 3. Anti-HIV-1 Activity and Cytotoxicity in Vitro

Several studies have described the in vitro anti-HIV activities and cytotoxicity of GRFT (Table 2) [5,25,26,27,28,29,30,31,32]. Mori et al. reported potent, sub-nanomolar antiviral efficacy of both native and recombinant GRFT against laboratory strains and primary isolates of T- and M- tropic HIV-1 [5]. In their publication, they observed that GRFT inhibited soluble gp120 from binding to CD4 receptor-expressing cells [5]. Cell-to-cell fusion and transmission of HIV-1 infection were also blocked by GRFT at similar concentrations [5]. In addition, the coadministration of monosaccharides such as glucose, mannose, and *N*-acetyl glucosamine hindered glycosylation-dependent binding of GRFT to soluble gp120 [5]. In parallel with this observation, Emau et al. also demonstrated that GRFT could block the infectivity of CXCR4- and CCR5-tropic HIV viruses at picomolar concentrations. They also confirmed the long-term stability of GRFT in a cervical/vaginal lavage [27]. In order to harness the HIV-inactivating power of GRFT, a new peptide called grifonin-1 was designed based on the three carbohydrate-binding amino acid sequences of GRFT. Its sub-micromolar anti-HIV activity was confirmed using an in vitro TZM-bl cell line and p24 gag antigen release assays [24]. In addition to its ability to suppress HIV-1 transmission in CD4+ T-lymphocytes, GRFT also subverted DC-SIGN (dendritic cell-specific intercellular adhesion molecule-3-grabbing nonintegrin)-mediated HIV capture [30]. Mechanistically, a binding competition between GRFT and gp120 for DC-SIGN was proposed as a potential mode of action for GRFT-mediated inhibition of HIV-1 capture by DC-SIGN [21]. Corresponding to this DC-SIGN-dependent antiviral activity, GRFT also potently inhibited giant cell formation between persistently HIV-infected T cells and noninfected CD4+ target T cells. This led to the suppression of HIV transmission, CD4+ T-cell destruction, and ultimately viral replication through the DC-SIGN mediated pathway [33]. This efficient blockage of the binding of DC-SIGN to immobilized gp120 by GRFT was further confirmed [21]. This observation was in harmony with the GRFT-mediated expulsion of gp120 from the gp120/DC-SIGN complex [21]. Interestingly, this highly potent inhibition of DC-SIGN-mediated capture and transmission by GRFT was markedly impaired when GRFT was mutated in one of its three carbohydrate-binding sites (D30A, D70A, or D112A) [21], further implicating its carbohydrate-binding sites as critical determinants for anti-HIV-1 activity. Regarding its cytotoxicity, two studies demonstrated that the CC_50_ concentration for GRFT was several hundred nanomolar [5,27], thousands of times higher than its reported antiviral concentrations.

## 4. Drug Combination

Four studies have described the combination effects of GRFT with either current anti-HIV drugs or potential therapeutics under development (Table 3) [26,28,29,32]. GRFT showed synergistic antiviral activity with tenofovir, maraviroc (a CCR5 antagonist), and enfuvirtide (a gp41 fusion peptide inhibitor) [29]. The different glycosylation patterns on the viral envelope of clade B and clade C gp120 had no observable effect on their synergistic antiviral action [29]. Covalently linking GRFT to the gp41-binding peptide C37 also exhibited a potency several-fold greater than that of GRFT alone in inhibiting HIV Env-mediated fusion in a CCR5-tropic cell-cell fusion assay [32]. In line with this observation, all GRFT/antiretroviral drug (entry inhibitors, reverse transcriptase inhibitors, integrase inhibitors, and protease inhibitors) combinations displayed either synergistic or additive effects in inhibiting cell–cell fusion and protected against target CD4+ T cell destruction [33]. GRFT/antiretroviral combinations also potently inhibited short-term viral replication in T-cells via DC-SIGN-mediated transmission [33]. Combinations of GRFT and other carbohydrate-binding agents (CBAs) including *Hippeastrum* hybrid agglutinin, *Galanthus nivalis* agglutinin, a mannose-specific monoclonal antibody (mAb) (2G12), microvirin, and banana lectin also showed synergistic activity against HIV-1, HIV-2, and even against certain CBA-resistant HIV-1 strains [28]. None of the CBAs competed with each other’s glycan-binding sites on gp120 since they have distinct binding patterns on the gp120 envelope [28]. In addition to antiviral synergy, gp120-GRFT complexes showed higher immunogenicity than the individual proteins per se. This suggests that removing the mannose moieties on monomeric gp120 improves the humoral immune response to this protein [34].

## 5. Anti-HIV-1 Mechanism of Action 

Six different antiviral mechanisms of action against HIV-1 by GRFT have been proposed (Table 4) [26,30,34,35,36,37]. A carbohydrate binding-dependent antiviral mechanism of GRFT was explored by using HIV-specific neutralizing monoclonal antibodies (mAbs) such as 2G12, 48d, b12, and b6 [25,26]. GRFT preferentially inhibited gp120 from binding to the 2G12 mAb, which targets N-linked glycans at positions 332, 339, and 392 on gp120. This suggests an overlapping binding specificity with the 2G12 mAb [25]. In addition, GRFT increased the interaction between gp120 and the 48d mAb, which recognizes a CD4-induced epitope [25]. GRFT also enhanced the binding of HIV-1 to plates coated with b12 and b6 mAbs [26]. This indicates that the binding of GRFT to gp120 triggers the exposure of the CD4-binding site on gp120. In particular, the glycan at position 386, which shields the CD4 binding domain of gp120, is also involved in the GRFT-mediated binding enhancements and the neutralization synergy between GRFT and b12 [26]. In addition, a synergy between GRFT and b12 was also exhibited when HIV-1 isolates became more sensitive to neutralization upon increasing HIV-1 binding of GRFT to b12 and b6 mAbs [26]. Together, these data suggest that GRFT-mediated blockage of a post-CD4 receptor binding event, such as the coreceptor binding with gp120, might be another plausible mechanism through which GRFT inhibits HIV-1 infection [26]. Since the glycans on HIV-1 gp120 play an important role in shielding neutralization-sensitive epitopes from antibody recognition [26], disruption of the mannose molecules on gp120 by GRFT may also increase antibody-dependent neutralization of HIV-1 particles. 

Although the multivalent interaction of GRFT with high-mannose oligosaccharides is believed to account for most of its picomolar antiviral potency, the looser correlation between gp120-binding ability and HIV inhibitory potency for the binding site mutants of GRFT suggests the possibility of another unknown antiviral mechanism of GRFT that is not based on simple gp120 binding [21]. According to an isothermal titration calorimetry binding study, GRFT bound to glucose and *N*-acetyl glucosamine in a similar fashion to that of mannose, demonstrating its flexible specificity in binding carbohydrates [23]. This binding flexibility might have an implication for its broad antiviral spectrum. To study the potential role of the GRFT dimer in the suppression of HIV-1 infectivity, either two or four amino acids were inserted at the dimerization interface of GRFT. This resulted in a monomeric form of GRFT (mGRFT) with greatly reduced antiviral activity against HIV-1. These results further emphasize the importance of multivalent interactions between dimeric GRFT and oligosaccharides present on HIV envelope glycoproteins for the successful cross-linking and aggregation of viral particles [20]. Interestingly, an obligate dimer of GRFT with a peptide linker between the two subunits altered the structure of gp120 by exposing the CD4 binding site. However, GRFT-linker-GRFT with mutated carbohydrate-binding sites largely lost this ability [37]. On the other hand, the glycan-specific DC-SIGN receptor binds the virus and mediates its transfer to CD4+ cells [38]. In this regard, GRFT’s ability to partially block gp120 from binding to human DC-SIGN [34] and its potent inhibition of DC-SIGN-dependent transfer of HIV-1 [38] could synergize with its antiviral action by blocking viral entry. To maximize the antiviral synergy caused by GRFT multimerization, tandem repeats of mGRFT (mGRFT tandemers) were engineered. They displayed picomolar-level antiviral activity in whole-cell anti-HIV assays [37]. However, since mGRFT tandemers could not aggregate HIV virions, Moulaei et al. suggested the intra-virion crosslinking of HIV envelope glycoproteins may be more integral to their antiviral activity [36]. Inter-virion aggregation or clustering of HIV-1 gp120 on the viral membrane was found to be related to neutralization potency [35]. 

## 6. Resistance

Five studies have characterized viral resistance profiles caused by chronic GRFT treatment (Table 5) [25,26,31,39,40]. Since the antiviral activity of GRFT mainly depends on disrupting the biological functions of multiple mannose molecules on viral glycoproteins, a reduction in the glycosylation levels of a target protein can lead to GRFT resistance. For example, the 234 and 295 glycosylation sites are involved in GRFT-induced HIV-1 neutralization since a concomitant lack of glycans at both positions resulted in natural GRFT resistance [25]. Conversely, introducing glycosylation sites at N234 and N295 in HIV-1 clade C virus increased GRFT antiviral potency [25]. In line with these observations, deglycosylation at position 295 or 448 decreased the sensitivity of a single transmitter/founder HIV Env to GRFT [31]. Since N295 and N448 are GRFT-specific, high-mannose, N-linked glycosylation sites on gp120, a single deglycosylation at N295 or N448 in primary or T-cell-line-adapted HIV-1 isolates also resulted in marked resistance to GRFT [40]. Furthermore, glycosylation sites at positions 230, 234, 241, and 289 located in the C2 region and 339, 392, and 448 in the C3–C4 region were also implicated in GRFT resistance [39]. A loss of glycosylation sites on gp120 as well as a rearrangement of glycans in V4 also led to HIV-1 subtype C resistance against GRFT [39]. In the case of DC-SIGN-dependent antiviral activity of GRFT, the effects of extra glycosylation seem to be dependent on the location of the glycosylation. For example, the introduction of the 234 glycosylation site abolished HIV-1 sensitivity to lectin’s ability to inhibit binding to DC-SIGN and virus transfer [38]. However, the addition of the 295 glycosylation site enhanced the inhibition of DC-SIGN-dependent HIV-1 transfer by GRFT [38]. Given the overlapping nature of the binding specificity displayed by GRFT and neutralizing antibodies against HIV-1, GRFT resistance could also affect HIV-1 sensitivity to antibody-dependent neutralization.

## 7. Toxicity

The ubiquitous nature of glycosylated proteins in the body raises concerns that GRFT may cause toxicity by interacting with glycosylated host proteins. For this reason, the effects of GRFT on host cells and animals have been studied extensively. According to five previous reports, GRFT does not exhibit any toxicity at its active antiviral concentrations (Table 6) [28,41,42,43,44,45]. GRFT induced only minimal changes in the secretion of inflammatory cytokines and chemokines by epithelial cells and human peripheral blood mononuclear cells (PBMCs) [43]. In addition, it had no measurable effect on cell viability or the levels of T-cell activation markers [43]. GRFT treatment induced only minimal alterations in the gene expression profile of human ectocervical cells [43]. It also caused no significant cell death, mitogenicity, activation, or cytokine release in mouse PBMCs [44]. No obvious changes were observed in animal fitness, blood chemistry, or complete blood count parameters in GRFT-treated mice [44]. Interestingly, the PBMC-bound form of GRFT was still able to maintain its antiviral activity, raising the potential of its versatile in vivo antiviral activity [43]. Chronic intravaginal or systemic administration of 2 mg/kg of GRFT was also non-toxic in mice [44]. GRFT, when administered in gel form, was not associated with any changes in the rectal proteome [42]. An increased abundance of two common and beneficial microbial taxa after GRFT treatment was due to placebo formulations and not to GRFT, itself [42]. This association between the placebo gel and changes in the rectal proteome and microbiota indicates the need to alter the components of the placebo gel in future studies [42]. GRFT was also well tolerated after subcutaneous administration in guinea pig and mouse models [41]. In addition, GRFT was found to be non-irritating and non-inflammatory in human cervical explants and in an in vivo rabbit vaginal irritation model [45]. In this study, no mitogenic activity was reported in cultured human lymphocytes treated with GRFT [45]. However, following GRFT treatment, reversible splenomegaly was observed with the activation of certain spleen B and T cells [44]. This GRFT-associated increase in spleen and liver mass was also noted in another study [41]. Therefore, an immune response elicited by GRFT treatment should be controlled in order to avoid potential GRFT immune-related toxicity [41].

## 8. Pharmacokinetic and in Vivo Efficacy Studies

Three studies were conducted to examine the in vivo antiviral efficacy and pharmacokinetic behavior of GRFT using various small animal models (Table 7) [41,46,47]. Subcutaneous injections of GRFT into guinea pigs and mice were very well tolerated, resulting in the accumulation of GRFT up to relevant therapeutic concentrations [41]. The serum from GRFT-treated animals was found to retain antiviral activity against HIV-1-enveloped pseudoviruses in a cell-based neutralization assay [41]. In addition, active GRFT, which is capable of neutralizing HIV-Env pseudoviruses, was also detected in rat fecal extracts after chronic oral dosing [46]. The in vivo efficacy of GRFT was also demonstrated in the humanized bone marrow-liver-thymus mice which were protected from vaginal infection with HIV-1 after being treated with recombinant *C. crescentus* expressing GRFT [47]. However, GRFT was not orally bioavailable even after chronic treatment [46]. 

## 9. Large-Scale Production

The clinical application of protein drugs requires a cost-effective large-scale production procedure to meet the high volume needed in a clinical setting. For efficient GRFT production, seven different production methods have been developed and optimized (Table 8) [45,48,49,50,51,52,53]. Giomarelli et al. used a fermenter and a rich, auto-inducing medium, which led to an approximately 45-fold increase in the total amount of GRFT per liter with approximately 70% of the protein expressed in the soluble fraction [49]. O’Keefe et al. were able to produce GRFT in multigram quantities by using *Nicotiana benthamiana* plants transduced with a tobacco mosaic virus vector expressing GRFT [45]. Hahn et al. employed a simple spraying method to introduce agrobacterium vectors into Nicotiana plants in the presence of a surfactant as a substitute for vacuum inoculation [50]. They found that recombinant GRFT is stable during the storage of plant biomass as silage, which is suitable for mass production of cost-sensitive products [50]. Fuqua et al., however, developed a simplified GRFT purification method [48]. They achieved >99% pure GRFT by generating the initial green juice extract in pH 4 buffer, heating the extract to 55 °C, incubating it overnight with a bentonite MgCl_2_ mixture, and purifying it via chromatography [48]. Vamvaka et al. successfully produced GRFT by using the endosperm of transgenic rice plants (*Oryza sativa*) [52]. They found that both crude and pure GRFT had potent anti-HIV activity, and the crude extracts were not toxic in human cell lines [52]. This suggests that crude GRFT with minimal processing could be administered to reduce costs associated with purification [52]. Petrova et al. expressed GRFT in the probiotic strains *Lactobacillus rhamnosus* GG and *L. rhamnosus* GR-1 for gastrointestinal and vaginal mucosal delivery, respectively [51]. Hoelscher et al. used stably transformed tobacco chloroplasts to produce GRFT, which accounted for up to 5% of the total soluble protein of the plant [53]. They also found that the tobacco, when dried, provides a storable source of GRFT that can be purified at a later date [53]. However, when produced in *Nicotiana benthamiana*, Kim et al. found that GRFT had pathologic effects on plants because it was directed to the apoplast during production, resulting in necrotic symptoms associated with hypersensitive response (HR)-like cell death [54]. They found that a specific interaction between GRFT and an apoplast-located endogenous glycoprotein, XYL1, initiated the HR response. To suppress GRFT-induced cell death in *Nicotiana benthamiana,* exogenous expression of naphthalene hydroxylase was suggested by the authors [54]. To avoid the emergence of HIV-1 strains resistant to single microbicides, Vamvaka et al. expressed components of multiple anti-HIV proteins including GRFT, 2G12 mAb, and cyanovirin-N in rice endosperm [55]. They found that extracts from plants expressing all three proteins showed enhanced in vitro binding to gp120 and synergistic HIV-1 neutralization [55]. Unexpectedly, they also found that synergistic HIV neutralization caused by the triple microbicide was enhanced by production in rice endosperm because rice globulin protein enhances gp120 binding in the expressed proteins [55]. 

## 10. A Formulation for Efficient Delivery

Protein stability is critical for long-term maintenance of pharmacological activity. For this reason, the susceptibility of GRFT to proteolytic digestion should be considered. GRFT was resistant to digestion by eight different proteases including pepsin, papain, leucine aminopeptidase, pronase, α-chymotrypsin, proteinase K, endoproteinase, and trypsin [56]. A number of different nanoparticle drug delivery systems have been tested to provide sustained and controlled delivery of GRFT, improve its solubility, protect its payloads, and enhance its mucosal permeability (Table 9) [57,58,59,60]. PLGA (poly lactic-co-glycolic acid) nanoparticles with a diameter of approximately 180–200 nm were successfully used in the co-delivery of GRFT and dapivirine in vitro [60]. Both drugs showed a biphasic release with an initial burst phase followed by a sustained release phase [60]. Grooms et al. successfully generated GRFT-modified electrospun fibers to inactivate HIV prior to cellular entry [57]. Furthermore, Lal et al. developed a self-administered, vaginal fast-dissolving insert (FDI) produced by freeze-drying that delivered safe and effective amounts of GRFT and carrageenan (GC), a sulfated polysaccharide extracted from seaweed [58]. Fibers comprised of methoxy polyethylene glycol-PLGA: poly n-butyl acrylate-co-acrylic acid (mPEG-PLGA: PBA-co-PAA) were able to achieve high GRFT loading. These GRFT-loaded fibers were well maintained within a simulated vaginal fluid (SVF) and showed pH-dependent release upon exposure to buffered SVF and simulated semen solutions [59]. 

## 11. Clinical Study

To date, two phase-one clinical studies have been initiated to investigate the potential toxicity of GRFT in healthy populations [61,62]. The first study aimed to evaluate the safety of GRFT in a CG gel used vaginally for a single dose and then for 14 consecutive days in healthy women [61]. This was a two-part study with the first part consisting of a single-dose and an open-label design, and the second part consisted of a multiple-dose, randomized, placebo-controlled study design. During the second part, 30 subjects were administered a placebo and 30 subjects were given the GRFT gel. In this study, the pharmacokinetic behavior of GRFT was also analyzed by forming a time-concentration curve of GRFT in blood samples. However, rising dose tolerance was not studied because of the minimal systemic absorbance of GRFT, which was reported in preclinical studies. Although the official study results are not available yet, the Population Council website reported GRFT to be safe for vaginal use for up to 14 days with potent anti-HIV activity in cell-based assays and cervical explants up to 8 h after receiving the dose [63]. In 2014, another phase-one clinical study for GRFT started as an integrated preclinical/clinical program. This program, which was named PREVENT (pre-exposure prevention of viral entry), aimed to provide a comprehensive set of data to facilitate an informed decision on whether GRFT should progress within the topical microbicides pipeline [62]. This was a randomized, double-blind phase-one safety and pharmacokinetic study of GRFT enema administered rectally to HIV-1 seronegative adults who practice URAI. The number and frequency of adverse events, the blood concentration of GRFT, and changes in humoral antibody response were analyzed. This on-going clinical study will be completed in 2021.

## 12. The Antimicrobial Activity of GRFT on Other Viruses

GRFT not only displays antiviral activity against HIV but also for other enveloped viruses such as SARS-CoV [10], MERS-CoV [11], HCV [12,13], HSV [14,15,64], JEV [16,17], and PEDV [18]. Even human papillomavirus (HPV), which is a non-enveloped virus, was inactivated by GRFT via a glycosylation-independent mechanism [15]. GRFT specifically bound to the SARS-CoV spike glycoprotein and inhibited viral entry [10]. GRFT also inhibited particles pseudotyped with the MERS-CoV spike protein from entering host cells [11]. Preincubation of HCV particles with GRFT prevented infection of Huh-7 hepatoma cells [13]. Furthermore, GRFT was able to interfere with the direct cell-to-cell transmission of HCV [13]. This anti-HCV activity was further demonstrated in vivo when HCV infection was mitigated in chimeric mice [12]. In this report, GRFT was readily bioavailable after subcutaneous injection, and it showed significant in vivo efficacy by reducing HCV viral titers in a mouse model system with engrafted human hepatocytes [12]. In contrast to HIV, HCV resistance to GRFT was not directly conferred by mutations in the envelope protein genes, but it could occur through an indirect mechanism involving mutations in other viral proteins [65]. GRFT displayed modest inhibitory activity against HSV-2 if it was present during viral entry, but if it was present post-entry, it completely blocked plaque formation, reduced plaque size, and prevented cell-to-cell propagation [14]. These in vitro findings translated into significant protection against genital herpes in mice treated with a 0.1% griffithsin gel [14]. The in vivo anti-HSV activity of GRFT was further demonstrated when murine model test subjects were protected by *C. crescentus* expressing GRFT after intravaginal infection with HSV-2 [64]. Levendosky et al. explored the antiviral properties of a combination product composed of GRFT and CG against HSV-2 and HPV. They found that GRFT was able to block the entry of HSV-2 and HPV into target cells but not the adsorption of HSV-2 and HPV onto target cells [15]. This GRFT/CG combination was also tested as a freeze-dried, FDI formulation. This product protected rhesus macaques against a high-dose vaginal simian HIV challenge 4 h after FDI insertion [66]. Furthermore, this GRFT/CG FDI also protected mice, vaginally, against HSV-2 and HPV pseudovirus [66]. In vitro experiments showed that the treatment of JEV with GRFT before inoculation into BHK- 21 cells inhibited infection in a dose-dependent manner [17]. In vivo experiments showed that GRFT (5 mg/kg) administered intraperitoneally before virus infection prevented mortality in mice challenged intraperitoneally with a lethal dose of JEV [17]. With regard to its antiviral mechanism, GRFT was shown to bind to JEV glycosylated viral proteins, specifically the enveloped and pre-mature glycoproteins [16]. In addition, GRFT was able to reduce PEDV infection in Vero cells [18]. 

## 13. Conclusions

Due to its novel carbohydrate-targeting antiviral mechanism of action, superior anti-HIV-1 efficacy, excellent host toxicity profile, synergistic interaction with other antiretroviral drugs, optimized large-scale production methodology, and various formulation methods for its efficient delivery, GRFT holds great promise as the first topical protein-based anti-HIV pre-exposure prophylactic. Even though a potentially adverse immunotoxicity issue observed during preclinical animal studies needs to be resolved, its broad antiviral spectrum, which is applicable to other enveloped viruses, could make GRFT the first universal antiviral therapeutic that can specifically target virus carbohydrates. Favorable outcomes from two ongoing phase-one clinical trials will accelerate the drug development process. If approved, GRFT will provide a superior way to prevent many transmissible viral infections.

## Figures and Tables

**Table 1:** Five structural studies involving GRFT. The structure resolution of GRFT and its major characteristics are presented.

Structure Studied	Crystal Resolution (A°)	Structural and Functional Characteristics	Ref.
GRFT alone or with mannose	1.3 and 0.94	A domain-swapped dimer, a Jacalin-related lectin with a β-prism motif, and three identical mannose-binding sites on each monomer	[19]
GRFT with glucose or *N*-acetylglucosamine	30–1.50 and 30–1.56	All six monosaccharide binding sites of GRFT are occupied by both glucose and *N*-acetylglucosamine with a mode of binding similar to that of mannose	[23]
GRFT with 1-6α-mannobiose or maltose	2.0 and 1.5	The binding of 1-6α-mannobiose is similar to that of mannose and the binding of maltose is weaker than that of mannose	[22]
Monomeric GRFT	30–0.97	Reduced activity against HIV-1 due to a loss of multivalent interaction and the binding of a monomeric GRFT to two different nona-mannosides	[20]
GRFT with a disrupted carbohydrate-binding site	NMR study	Reduced binding to mannose and a weaker correlation between anti-HIV-1 activity and gp120 binding	[21]

**Table 2:** Anti-HIV-1 activity and cytotoxicity of GRFT in vitro. N/A indicates “not applicable.”

HIV-1 Type	Cell Line	Anti-HIV-1 Assay Readout	EC_50_ (nM)	CC_50_ (nM)	Ref.
Laboratory strains and primary isolates of T- and M-tropic HIV-1	CEM-SS cells, PBMC, and macrophage	Virus-induced cell killing	0.043–0.63	>783	[5]
CXCR4-and CCR5-tropic HIV-1, SHIV strains, and SIVmac251	CEM-174, MT-2, MT-4, and PBMC	p24	<1	>500	[27]
HIV-1 subtype C primary virus isolates	TZM-bl and PBMC	TZM-bl and p24	0.4	N/A	[25]
HIV-1 CCR5-tropic strain ADA and CXCR4-tropic strain HXB2	HeLa-ADA effector cells and HeLa-P5L CCR5-bearing target cells	CCR5-tropic cell-cell fusion	1.31	N/A	[32]
HIV-1 clade B and clade C isolates	PBMCs and CD4+ MT-4 cells	p24 HIV-1 core Ag ELISA	0.18	N/A	[29]
HIV-1 subtype B QH0515 and C SW7 and Du179	Raji/DC-SIGN cells and TZM-bl cells	HIV-1 binding to the DC-SIGN receptor	345	N/A	[26]
HIV-1 subtype B QH0515 and C SW7 and Du179	Raji/DC-SIGN cells and TZM-bl cells	HIV-1 bound Raji cells transferred to CD4 cells	4.8–35	N/A	[26]
HIV-1 X4 strain IIIB and dual-tropic R5/X4 HIV-1 strain HE	Persistently HIV-infected T cells and noninfected CD4+ target T cells	Giant cell formation	0.087	N/A	[28]
HIV-1 X4 strain IIIB and dual-tropic R5/X4 HIV-1 strain HE	Raji.DC-SIGN cells	DC-SIGN mediated HIV transmission	0.025	N/A	[28]
HIV-1(IIIB) and HIV NL4.3	CEM, C8166, HuT-78, and Sup-T1 cells, Raji/DC-SIGN cells	DC-SIGN-mediated virus capture	1.5	N/A	[30]
HIV-1(IIIB) and HIV NL4.3	CEM, C8166, HuT-78, and Sup-T1 cells, Raji/DC-SIGN cells	HIV-1 transmission to CD4+ T-lymphocytes	0.012	N/A	[30]
Single transmitted/founder HIV-1	TZM-bl cells	Neutralization assay using pseudotyped viruses	0.006–10	N/A	[31]

**Table 3:** Drug combination results with GRFT.

Cell Line	Assay Readout	Combination Drug	Result	Ref.
PBMCs and CD4+ MT-4 cells	p24 HIV-1 core Ag ELISA	Tenofovir, maraviroc, and enfuvirtide	Synergy	[29]
HeLa-ADA effector cells and HeLa-P5L CCR5-bearing target cells	CCR5-tropic cell-cell fusion	Covalently linked gp41-binding peptide C37	Synergy	[32]
TZM-bl cells, U87-CCR5 and U87-CXCR4.	Pseudovirus neutralization	2G12 mAb	Synergy	[26]
Persistently HIV-infected T cells and noninfected CD4+ target T cells	Giant cell formation	Efavirenz, tenofovir, raltegravir, and elvitegravir	Synergy	[28]
Raji.DC-SIGN cells	DC-SIGN mediated HIV transmission	Tenofovir, saquinavir, and 2G12 mAb	Synergy	[28]
MT-4 cells, PBMC	Virus-induced cytopathic effect	Microvirin, 2G12 mAb, BanLec, and HHA	Synergy	[28]

**Table 4:** GRFT anti-HIV-1 mechanisms of action.

The Effect of GRFT on HIV-1 gp120	Ref.
Exposure of the CD4 binding site of gp120 through the glycan at position 386 and blockage of coreceptor binding step	[26]
Inhibition of mannose-binding to gp120 and improvement of the humoral immune response to gp120	[34]
Inhibition of gp120 binding to DC-SIGN and expulsion of gp120 from the gp120/DC-SIGN complex	[30]
Alteration of gp120 structure through the exposure of the CD4 binding site	[37]
Intra-virion crosslinking of gp120	[36]
Inter-virion aggregation or clustering of gp120	[35]

**Table 5:** HIV-1 resistance mechanisms against GRFT.

HIV Type	Cell Line	Assay Readout	Resistance Mechanism	Ref.
HIV-1 subtype C primary virus isolates	TZM-bl and PBMC	HIV-1 neutralization assay	Loss of glycans at positions 234 and 295	[25]
Primary and T-cell-line-adapted HIV-1 isolates	TZM-bl cells	Env-pseudotyped viruses to infect TZM-bl cells	Loss of glycan at position 295 and 448	[40]
HIV-1 subtype C primary virus isolates	Raji/DC-SIGN cells	The DC-SIGN-mediated HIV-1 transfer to TZM-bl cells	Introduction of a glycan at position 234	[38]
HIV-1 subtype C primary virus isolates	TheTZM-bl and PBMC	HIV-1 neutralization assay in PBMC	Loss of glycan at positions 230, 234, 339, 392, and 448	[39]
Single transmitted/founder HIV-1	TZM-bl cells	Neutralization assay using pseudotyped viruses	Loss of glycan at positions 295, 339, and 448	[31]

**Table 6:** Toxicity studies of GRFT in various cell and animal models.

Models Used	Dose Tested	Effects of GRFT	Ref.
Human cervical explants and rabbits	Up to 2 μM	No effect on the production of proinflammatory cytokines and chemokines. No vaginal irritation in rabbits.	[45]
Human cervical epithelial cells, cervicovaginal cells, and PBMCs	Up to 84 μM	Minimal changes in secretion of inflammatory cytokines and chemokines No measurable effect on cell viability and T-cell activation markers.	[43]
Guinea pig BALB/c mice	Single 50 mg/kg and 10 daily subcutaneous injections of 10 mg/kg	Minimal overall toxicity. Well tolerated. Increase in spleen and liver mass.	[41]
Mouse PBMC and BALB/c mice with parenteral administration	Up to 4 μM in vitro, subcutaneously with a single dose of 10 mg/kg, fourteen daily doses of 10 mg/kg, 2 mg/kg subcutaneous, intravaginal, and intraperitoneal administration	No mitogenic properties in vitro. No effect on cell surface activation markers or animal fitness. No major organ toxicity with reversible splenomegaly.	[44]
Rhesus macaques	Intravaginal 0.1% gels	No change in rental proteome or microbiome	[42]

**Table 7:** In vivo anti-HIV-1 activity of GRFT in animal models.

Models Used	Dose Tested	Effects of GRFT	Ref.
Guinea pig BALB/c mice	Single 50 mg/kg and 10 daily subcutaneous injections of 10 mg/kg.	Retention of antiviral activity in serum	[41]
Sprague Dawley (SD) rats	A single dose of 10 mg/mL intravenously or subcutaneously. Ten 40 mg/kg doses for 10 days.	Neutralization activity found in fecal extracts	[46]
Humanized bone marrow-liver-thymus mice	10^8^ GRFT-expressing recombinant *C. crescentus* intravaginally.	Protection against HIV-1 infection	[47]

**Table 8:** Large-scale GRFT production methods. N/A indicates “not applicable.”

Production Method	Expression Organism	Effects and Yield	Ref.
Transformation and the use of an autoinduction fermentor	*Escherichia coli*	45-fold increase	[49]
Transduction with tobacco mosaic virus	*Nicotiana benthamiana*	Multigram quantity	[45]
Agrobacterium vectors	*Nicotiana benthamiana*	90% of the leaf cells and 50% of the total soluble protein	[50]
Transduction with tobacco mosaic virus in pH 4 buffer, heating the extract to 55 °C, a bentonite MgCl_2_ mixture, and chromatography.	*Nicotiana benthamiana*	88% ± 5% of griffithsin from the initial extract	[48]
Particle bombardment	Rice endosperm	223 μg/g dry seed weight	[52]
Use of probiotic microorganisms	*Lactobacillus rhamnosus GG* and *L. rhamnosus GR-1*	N/A	[51]
Chloroplast transformation	*Nicotiana tabacum*	360 μg of pure griffithsin per gram	[53]

**Table 9:** Pharmaceutical formulations for the efficient delivery of GRFT.

Formulation Method	Delivery Route	Effects on Delivery	Ref.
PLGA nanoparticles	Vaginal	A biphasic release with an initial burst phase followed by a sustained release phase	[60]
Electrospun fibers	In vitro	Maintenance of antiviral efficacy	[57]
FDI comprised of	Vaginal	Good friability, hardness, and stability	[58]
mPEG-PLGA:PBA-co-PAA	Vaginal	High GRFT loading and pH-dependent release	[59]
